# A cross-sectional study of obesogenic behaviours and family rules according to family structure in European children

**DOI:** 10.1186/s12966-020-00939-2

**Published:** 2020-03-05

**Authors:** Katharina Stahlmann, Antje Hebestreit, Stefaan DeHenauw, Monica Hunsberger, Jaakko Kaprio, Lauren Lissner, Dénes Molnár, Alelí M. Ayala-Marín, Lucia A. Reisch, Paola Russo, Michael Tornaritis, Toomas Veidebaum, Hermann Pohlabeln, Leonie H. Bogl

**Affiliations:** 1 Leibniz Institute for Prevention Research and Epidemiology – BIPS, Bremen, Germany; 2 Department of Public Health, Ghent University, Ghent, Belgium; 3 School of Public Health and Community Medicine, Institute of Medicine, Sahlgrenska Academy, University of Gothenburg, Gothenburg, Sweden; 4 Institute of Molecular Medicine FIMM, University of Helsinki, Helsinki, Finland; 5 Department of Public Health, University of Helsinki, Helsinki, Finland; 6 Department of Paediatrics, Medical School, University of Pécs, Pécs, Hungary; 7 GENUD (Growth, Exercise, Nutrition and Development) Research Group, Faculty of Health Sciences, University of Zaragoza, Zaragoza, Spain; 8 Department of Management, Society and Communication, Copenhagen Business School, Frederiksberg, Denmark; 9 Institute of Food Sciences, National Research Council, Avellino, Italy; 10 Research and Education Institute of Child Health, Strovolos, Cyprus; 11 Department of Chronic Diseases, National Institute for Health Development, Tallinn, Estonia; 12 Department of Epidemiology, Center for Public Health, Medical University of Vienna, Vienna, Austria

**Keywords:** Lifestyle, Family types, Blended families, Single-parent, Family rules

## Abstract

**Background:**

There has been an increase in children growing up in non-traditional families, such as single-parent and blended families. Children from such families have a higher prevalence of obesity and poorer health outcomes, but research on the relationship with obesogenic behaviours is limited.

**Objectives:**

Therefore, the aim of this study was to investigate whether there are associations between family structures and obesogenic behaviours and related family rules in European children and adolescents.

**Methods:**

The sample included 7664 children (mean age ± SD: 10.9 ± 2.9) from 4923 families who were participants of the multi-centre I.Family study (2013/2014) conducted in 8 European countries. Family structure was assessed by a detailed interview on kinship and household. Obesogenic behaviours (screen time, sleep duration, consumption of sugar-sweetened beverages (SSBs)) and family rules (rules for computer and television, bedtime routine, availability of SSBs during meals) were determined by standardized questionnaires. Multilevel mixed-effects linear and logistic regression models were used to model the associations of family structure with obesogenic behaviours and family rules. Sex, age, parental education level, number of children and adults in the household and BMI z-score were covariates in the models. Two-parent biological families were set as the reference category.

**Results:**

Children from single-parent families were less likely to have family rules regarding screen time (OR: 0.62, 95% CI: 0.40–0.94, *p* = 0.026) with higher reported hours of screen time per week (β = 2.70 h/week, 95% CI: 1.39–4.00, *p* < 0.001). The frequency of weekly SSB consumption differed by family structure in a sex-specific manner: girls from single-parent (β = 3.19 frequency/week, 95% CI: 0.91–5.47, *p* = 0.006) and boys from blended/adoptive families (β = 3.01 frequency/week, 95% CI: 0.99–5.03, *p* = 0.004) consumed more SSBs. Sleep duration, bedtime routines and availability of SSBs during meals did not differ between children from these family structures. Parental education did not modify any of these associations.

**Conclusions:**

Parents in non-traditional family structures appear to experience more difficulties in restricting screen time and the intake of SSBs in their children than parents in traditional two-parent family structures. Our findings therefore suggest that additional support and effective strategies for parents in non-traditional families may help to reduce obesogenic behaviours in children from such family types.

## Introduction

Obesogenic behaviours, including high screen time [1, 2], consumption of sugar sweetened beverages (SSBs) [3, 4] and lack of sleep [5, 6] have become more prevalent in children and adolescents in recent years. In addition to age, socioeconomic status (SES), sex [7, 8] and parental education [7, 9], the family environment has a major influence on children’s and adolescent’s behaviour [10]. Parents often serve as role models for their children and their parenting style, including family rules and routines, may affect their children’s obesity-related behaviours [11–15].

As family structures in Western societies have changed during recent decades, the association of family structure and child health is of increasing interest to societies. In particular, there has been an increase in children growing up in non-traditional families, such as single-parent and blended families [16, 17]. Children from such families have often poorer general health outcomes [18–20] and a higher prevalence of obesity [21–23]. Although obesogenic behaviours could account for the association between family structure and obesity, most previous studies have focused merely on BMI/obesity as an outcome [9, 21–24]. The few studies that have focused on obesogenic behaviours indicate that screen time [25, 26] and the consumption of SSBs [25] is higher in children and adolescents from non-traditional families, whereas sleep duration is lower in adolescents from single-parent families than from two-parent families [27]. However, most of these studies were conducted in countries of Anglo-Saxon origin, where English is the dominant language. To our knowledge, only one study examined family structures and obesogenic behaviours in Europe [28].

We have previously reported using the present cohort that family structure, defined as either the number and type of cohabiting adults or the number of siblings, is an important determinant of childhood overweight or obesity [23, 29]. However, the current state of research lacks data regarding obesogenic behaviours and family rules in European children and adolescents from different family structures. Understanding the role of family structure in children’s and adolescent’s health behaviour in the European context is crucial in order to develop effective interventions that help to combat the obesity epidemic. Therefore, the aim of this study was to investigate the associations between family structure and obesogenic behaviours as well as family rules in children and adolescents from eight European countries.

## Methods

### Data

The present analyses were performed using data from the multi-centre I.Family study that was conducted between 2013 and 2014 in eight European countries (Belgium, Cyprus, Estonia, Germany, Hungary, Italy Spain and Sweden). As a follow-up of the children from the IDEFICS (Identification and prevention of dietary- and lifestyle-induced health effects in children and adolescents) study, the I.Family study re-examined 7117 of the originally studied 18,772 IDEFICS children (participated in either the baseline-survey T0 or the first follow-up T1). Since I.Family aimed at investigating entire families, another 2501 siblings were newly recruited in this further survey (T3, 4–6 years later) [30]. Children who had already participated at T0 or T1 were called index children. The I.Family study also aimed at including at least one parent, who provided detailed information on kinship and household. In total, 6167 families with an average of 2.0 children and 4.1 family members participated in the I.Family study. Ethical approval was obtained by each of the eight centres conducting the fieldwork from the appropriate local ethics committees. All children and their parents gave informed consent to participate in the study. Confidentially was ensured by a common data protection protocol approved by all survey centres.

### Family structure

The family structure was determined based on a detailed kinship and household interview [31]. The main household of the previously participating IDEFICS children was identified and a computer-assisted telephone or computer-assisted personal interview conducted. The parent (or legal guardian) was asked how he/she is related to the oldest index child. The interviewer asked about the number of children and adults (aged ≥18 years) in the household, and further enquired about the relationship status to the chosen index child of each household member. The interviewer assigned codes for the relationship status that corresponded to ‘biological mother’, ‘biological father’, ‘biologically unrelated female adult’, ‘biologically unrelated male adult’, ‘any other adult’, ‘biological sibling’, ‘half-sibling’ or ‘non-biological sibling’ of the chosen index child.

Based on this information, we derived the variable family structure that distinguished between (a) two-parent biological families (the child lives with both biological parents and possibly other adults, either as an only child or with full siblings), (b) single-parent families (the child lives with one biological parent or step-parent, either as an only child or with full-, half-, or step-siblings), (c) two-parent blended/adoptive families (the child lives with one biological parent and one non-biological parent and possibly other adults, either as an only child or with the presence of full, half-, or step-siblings, (d) other family types (the child lives without parents but with other adult(s) such as second-degree relatives (e.g. grandparents, aunt, uncle), either as an only child or with full-, half-, or step-siblings).

### Primary outcome variables

Information on screen time, sleep duration and the consumption of SSBs was collected via a children’s questionnaire filled out by the parents for children 2–11 below the age of 12 years and a self-completed teen questionnaire for children aged 12 and over [32]. In order to assure high data quality and to improve standardisation in this multicentre study, all instruments including the questionnaires were pretested for their feasibility, robustness and acceptability [33].

Screen time was calculated by summing total use of audiovisual media on weekdays and weekends. The corresponding questions were: ‘How long do you/does your child usually watch TV and/or video/DVD per day?’ and ‘How long do you/does your child usually sit at a computer/game console per day? (Please disregard the time spent with internet-use.)’. Possible answer categories (recoded to hours per day), subdivided into weekdays and weekend days, were: not at all (recoded to 0), less than 30 min per day (recoded to 0.25), 30 min to 1 h per day (recoded to 0.75), about 1–2 h per day (recoded to 1.5), about 2–3 h per day (recoded to 2.5) and more than 3 h per day (recoded to 4). To derive total screen time in hours per week, the weekday estimates were multiplied by 5 and the weekend estimates by 2 and then summed.

Sleep duration was also derived from 2 questions: ‘What is the amount of time you sleep/your child sleeps during a 24-hour period on school days?’ and ‘What is the amount of time you/your child sleep(s) during a 24 hour period on weekends/vacations?’. Separate answers could be given for sleep at night and for daytime napping and both were summed in the calculation. To derive total sleep duration in hours per day, weekly frequencies were calculated first as described above for screen time, and then the total hours of sleep per week were divided by 7.

The consumption of SSBs was calculated based on the sum of 3 beverage questions from the child eating habits questionnaire including carbonated sugar-sweetened drinks (e.g. cola, lemonade, non-alcoholic beer, etc.), non-carbonated sugar-sweetened drinks (e.g. bottled ice tea, syrup-based drinks and similar, fruit juices with less than 100% fruit, sports drinks, non-alcoholic wine, etc.) and fruit juices (100% fruit or packaged orange juice, apple juice, etc.) [34]. In addition, local examples were given for each beverage item. The specific question was ‘In the last month, how many times did you/your child drink the following food items?’ and possible answer categories (recoded to times per week) were: never/less than once a week (recoded to 0), 1–3 times a week (recoded to 2), 4–6 times per week (recoded to 5), once per day (recoded to 7), twice per day (recoded to 14), three times per day or more (recoded to 21) and 4 or more times per day (recoded to 30). The consumption frequencies were then summed to derive the consumption of SSBs as frequencies per week. For those children and adolescents for whom more than half of the questions on beverage items in the child eating habits questionnaire were answered (that is, more than 6 beverage items out of a total of 12 beverage items), omitted items were treated as not consumed (recoded to 0) [35, 36]; else, the frequency of consumption of SSBs was not calculated (recoded to missing).

### Secondary outcome variables

One of the parents filled in a family questionnaire that addressed general questions regarding family rules and related behaviours that applied for the whole family [32]. One question enquired about the availability of SSBs at home during meal times by asking ‘Are sweetened soft drinks available at home during meals? (with local examples)’ with two response categories ‘yes, often or always’ and ‘no or rarely’. Two questions asked about the presence of rules on how much time the child is allowed to spend watching TV and on how much time the child is allowed to spend using the computer by asking ‘Do you have any rules about how much time your child/children can spend watching TV/on the computer?’ with two response categories ‘yes’ and ‘no’. These two questions were combined for the family rule variable on time spent watching TV or on computers. The family was considered to have family rules regarding screen time behaviours if either question was answered with ‘yes’. Bedtime routine was considered because it has previously been shown that a consistent nightly bedtime routine is beneficial in improving multiple aspects of sleep in children [37], and it was a key message in our previous IDEFICS multilevel obesity prevention on sleep [38]. The variable was derived from the children’s and teen questionnaires for each child separately by the question ‘Does you/ your child have a regular bedtime routine?’ with the dichotomous answer choices ‘yes’ or ‘no’. For 82.5% of the families, bedtime routines were similar for all children in the family. The family was considered to have rules regarding bedtime routines if at least one child in the family had routines.

### Covariates

We considered sex, age, parental education level, the number of children and adults in the household and children’s BMI z-score as covariates. Parent’s highest education level was classified according to the International Standard Classification of Education (in the following ISCED) for cross-country comparability into low, middle and high levels [39]. We considered two European regions: Belgium, Estonia, Germany and Sweden were considered as Northern and Hungary, Cyprus, Italy Spain as Southern European countries. The number of adults and children in the household was obtained from the kinship interview. Weight was measured by a TANITA digital scale (TANITA Europe GmbH, Sindelfingen, Germany) and height was measured using a portable stadiometer (Seca GmbH & Co. KG., Hamburg, Germany). Weight was measured to the nearest 0.1 kg and height to the nearest 0.1 cm. Measurements were performed in the morning, in light clothing and in fasting condition. Age and sex-specific BMI z-scores were computed according to Cole & Lobstein [40].

### Description of the analytic sample

We included all children aged 2 to 17 years with complete kinship information and for whom data on at least one primary outcome variable was available. Out of a total of 9618 children below the age of 18 years, we excluded individuals for whom the family structure was not assignable due to missing or incomplete information in terms of the relationship towards the child at the interview (*n* = 1451) and for whom all 3 primary outcome variables were missing (*n* = 503). Thus, the final sample consisted of 7664 children from 4923 families. The actual number of children for each analysis varied because individuals could consent to single components of the study while abstaining from others. Data on obesogenic behaviours was collected for each child/adolescent and therefore analysed on the level of the child (child-level analysis). Data on family rules for screen time and availability of SSBs during meals were reported by a parent for the whole family, and bedtime routines were mostly similar for all children in the family and therefore analysed at the level of the family (family-level analysis).

### Statistical analysis

Continuous variables are expressed as means and standard deviations (SD) and categorical variables as numbers and percentages. Differences in general characteristics between family structures were tested with ANOVA or the chi-square test, as appropriate. The data has a multilevel structure, where children are nested within families and within countries. At the child-level analyses, the outcome variables (obesogenic behaviour) were continuous and we used multilevel mixed-effects linear regression with family and country as random effects. At the family-level analyses, the outcome variables (family rules) were binary and we used mixed-effects logistic regression with country as a random effect. Model 1 included the age, sex, parental education and number of adults and children living in the household as covariates. Model 2 additionally included the child’s BMI-z-score. In all analyses, two-parent biological families were chosen as the reference category. For the family-level analyses, sex was categorised into families with ‘only girls’, ‘only boys’ or ‘both sexes’ and age was categorized into ‘younger (<12 years) children’, ‘older ( ≥12 years) children’ or ‘younger (<12 years) and older ( ≥12 years) children’ within the family. Model 2 additionally included the mean BMI-z-score of all children in the household. We further tested interactions with sex, age group (younger vs. older children), parental education level (low/middle vs. high educated) and European regions (Northern countries vs. Southern countries) and we stratified the results by sex, age group and parental education level. The *p*-values for interactions are not corrected for multiple testing and should be considered as explorative. All analyses were conducted with Stata 15 (Stata Corp, College Station, TX).

## Results

### Descriptive characteristics of the study population

The sample included 7664 children with a mean age of 10.9 years (SD: 2.9 years) and an about equal sex distribution (Table 1). Over 80% of the children lived in two-parent biological families, 10% in single-parent families, and 6% in blended/adoptive and less than 2% in other family types. Over half of the participating children had one sibling, while about one quarter of the children had two siblings. Half of the children had parents with high educational background. Most families had rules regarding screen time and routines regarding bedtime. SSBs were available during meals in about a quarter of the families.

**Table 1:** Descriptive characteristics of the children and families by family structure

Variable						*P*-value
	Two-parent biological	Single-parent	Two-parent blended/ adoptive	Other family types	Total	
**Information on the child-level**						
**Family structure, n (%)**	6293 (82.1)	756 (9.9)	482 (6.3)	133 (1.7)	7664	
**Age (years), mean ± SD**	10.8 ± 2.9	11.5 ± 2.6	11.3 ± 2.8	11.4 ± 2.3	10.9 ± 2.9	0.0001
**BMI z-score, mean ± SD**	0.59 ± 1.15	0.57 ± 1.16	0.57 ± 1.16	0.57 ± 1.16	0.58 ± 1.2	0.0006
**Sex, n (%)**						
Male	3194 (50.8)	376 (49.7)	239 (49.6)	74 (55.6)	3883 (50.7)	0.61
Female	3099 (49.2)	380 (50.3)	243 (50.4)	59 (44.4)	3781 (49.3)	
**Number of children in the household**						
1	560 (8.9)	225 (29.8)	64 (13.3)	50 (37.6)	899 (11.7)	0.0001
2	3456 (54.9)	370 (48.9)	182 (37.8)	55 (41.4)	4063 (53.0)	
3	1721 (27.4)	142 (18.8)	162 (33.6)	18 (13.5)	2043 (26.7)	
4	556 (8.8)	19 (2.5)	74 (15.4)	10 (7.5)	659 (8.6)	
**Country, n (%)**						
Italy	1387 (22.0)	58 (7.7)	23 (4.8)	22 (16.5)	1490 (19.4)	
Estonia	437 (6.9)	86 (11.4)	130 (27.0)	19 (14.3)	672 (8.8)	
Cyprus	1878 (29.8)	124 (16.4)	41 (8.5)	20 (15.0)	2063 (26.9)	
Belgium	111 (1.8)	19 (2.5)	11 (2.3)	0 (0)	141 (1.8)	
Sweden	605 (9.6)	86 (11.4)	46 (9.5)	1 (0.8)	738 (9.6)	
Germany	803 (12.8)	175 (23.2)	106 (22)	26 (19.6)	1110 (14.5)	
Hungary	779 (12.4)	165 (21.2)	114 (23.7)	38 (28.6)	1096 (14.3)	
Spain	293 (4.7)	43 (5.7)	11 (2.3)	7 (5.3)	354 (4.6)	< 0.0001
**Parent’s education level (ISCED), n (%)**						
Low	330 (5.5)	49 (6.8)	32 (6.8)	18 (14.3)	429 (5.8)	< 0.0001
Medium	2636 (34.6)	333 (45.9)	235 (49.9)	79 (62.7)	3283 (44.6)	
High	3068 (50.9)	344 (47.4)	204 (43.3)	29 (2.03)	3655 (49.6)	
**Screen time (hours/week), mean ± SD**	15.8 ± 10.1	18.6 ± 11.8	17.6 ± 12.1	17.1 ± 11.6	16.2 ± 10.5	< 0.0001
**Sleep duration (hours/day), mean ± SD**	9.4 ± 1.1	9.3 ± 1.1	9.4 ± 1.3	9.4 ± 1.1	9.4 ± 1.11	0.004
**SSBs (servings/day), mean ± SD**	1.0 ± 1.3	1.1 ± 1.6	1.3 ± 1.6	1.2 ± 1.5	1.1 ± 1.3	0.01
**Information on the family-level**						
**Family structure, n (%)**	3881 (78.8)	580 (11.8)	360 (7.3)	102 (2.1)	4923	
**Presence of rules for screen time, n (%)**						
Present	2983 (88.1)	402 (82.2)	248 (80)	77 (85.6)	3710 (86.8)	< 0.0001
Not present	404 (11.9)	87 (17.8)	62 (20)	13 (14.4)	566 (13.2)	
**Availability of SSBs at home, n (%)**						
Available	774 (22.2)	121 (24.2)	78 (24.6)	28 (30.4)	1001 (22.7)	0.17
Not available	2718 (77.8)	379 (75.8)	239 (75.4)	64 (69.6)	3400 (77.3)	
**Presence of bedtime routines, n (%)**						
Present	2101 (82.2)	245 (81.9)	163 (85.3)	41 (74.6)	2550 (82.2)	0.32
Not present	456 (17.3)	54 (18.1)	28 (14.7)	14 (25.5)	552 (17.8)	

Abbreviations: n, number of children; SD, Standard Deviation; BMI, body mass index; ISCED, International Standard Classification of Education; SSBs, sugar-sweetened beverages. *P* values are from ANOVA or chi-square test, as appropriate. The number of subjects for each variable might vary due to missing data

### Association between obesogenic behaviours and family rules

Obesogenic behaviours in children and adolescents were significantly related to the presence of family rules for the specific behaviours. In models adjusted for sex, age, parental education level, number of children and adults in the household, BMI z-score, education and family structure, children in families with rules regarding screen time watch significantly less television (β = − 2.5 h/day, CI: 3,33,-1.68, *p* < 0.001) than children who lived in families where such rules were absent (*p* < 0.001). Similarly, in fully-adjusted models, children with bedtime routines slept significantly more (β = 0.11 h/day, CI: 0.04, 0.18, *p* = 0.002) than children without bedtime routines and the availability of SSBs during meals was strongly related to children’s intake (β = 5.4 serving/week, CI: 4.6, 6.2, p < 0.001).

### Association between family structures and obesogenic behaviours

The associations between family structure and obesogenic behaviours in children are shown in Table 2. Children from single-parent families had higher screen times than those from two-parent biological families in a model adjusted for age, sex, parental education level, number of children and adults in the household and BMI z-score (β = 2.70 h/week, 95% CI: 1.39–4.00) (Table 2). Children from blended/adoptive families provided some evidence for having a higher screen time than two-parent biological families. Overall, no associations of family structure with the consumption of SSBs or sleep durations were seen.

**Table 2:** Association between family structure and obesogenic behaviours in European children and adolescents

	Model 1		Model 2	
	β (95% CI)	*P*-value	β (95% CI)	*P*-value
**Screen time (hours/week)**				
**Number of children**	7013		6987	
Two-parent biological	Reference		Reference	
Single-parent	2.81 (1.51,4.12)	< 0.001	2.70 (1.39,4.00)	< 0.001
Two-parent blended/adoptive	1.06 (0.01,2.12)	0.047	1.03 (−0.02,2.08)	0.054
Other family types	0.03 (−1.90,1.95)	0.98	0.08 (−1.86,2.02)	0.94
**Sleep duration (hours/day)**				
**Number of children**	7074		7048	
Two-parent biological	Reference		Reference	
Single-parent	−0.05 (− 1.18,0.08)	0.47	− 0.04 (− 0.17,0.09)	0.57
Two-parent blended/adoptive	0.004 (− 0.10,0.11)	0.93	0.002 (− 0.10,0.11)	0.96
Other family types	−0.004 (− 0.20,0. 19)	0.97	0.02 (− 0.18,0.21)	0.88
**SSBs (servings/week)**				
**Number of children**	4116		4096	
Two-parent biological	Reference		Reference	
Single-parent	0.95 (−0.72,2.62)	0.26	1.01 (−0.66,2.69)	0.23
Two-parent blended/adoptive	1.09 (−0.28,2.45)	0.12	1.05 (−0.32,2.41)	0.13
Other family types	0.61 (−1.84,3.06)	0.62	0.57 (−1.91,3.05)	0.65

Abbreviations: β, Beta coefficient; CI, Confidence Interval; SSBs, sugar-sweetened beverages

Model 1 is adjusted for sex, age, parental education level, number of children and adults in the household

Model 2 is additionally adjusted for BMI z-score

Results of the sex-stratified analyses are shown in Table 3. Girls and boys from single parent families had higher screen times than those from two-parent biological families, whereby the effect was stronger in girls (β = 2.99 h/week, 95% CI: 1.29–4.68) than in boys (β = 2.05 h/week, 95% CI: 0.23–3.87). The association between family structure and frequency of SSBs showed different association in boys vs. girls. Boys from two-parent blended/adoptive families (but not single-parent families) consumed more SSBs per week than boys from two-parent biological families, whereas girls from single-parent families (but not two-parent blended/adoptive families) consumed more SSBs per week than girls from two-parent biological families in fully-adjusted models (P_interaction_ = < 0.05). Sleep duration did not differ by sex between children from different family structures.

**Table 3:** Association between family structure and obesogenic behaviours in European boys and girls

	Boys				Girls			
	Model 1 Coef. (95% CI)	*P*-value	Model 2 Coef. (95% CI)	*P*-value	Model 1 Coef. (95% CI)	*P*-value	Model 2 Coef. (95% CI)	*P*-value
**Screen time (hours/week)**								
**Number of boys/girls**	3532		3516		3481		3471	
Two-parent biological	Reference		Reference		Reference		Reference	
Single-parent	2.15 (0.33,3.97)	0.021	2.05 (0.23,3.87)	0.027	3.12 (1.43,4.82)	< 0.001	2.99 (1.29,4.68)	0.001
Two-parent blended/adoptive	1.72 (0.17,3.27)	0.029	1.76 (0.22,3.31)	0.025	0.57 (−0.72,1.87)	0.39	0.47 (−0.83,1.76)	0.48
Other family types	0.54 (−2.18,3.27)	0.70	0.54 (−2.22,3.30)	0.70	−0.87 (−3.38,1.65)	0.50	−0.73 (−3.26,1.81)	0.58
**Sleep duration (hours/day)**								
**Number of boys/girls**	3583		3566		3491		3482	
Two-parent biological	Reference		Reference		Reference		Reference	
Single-parent	−0.09 (−0.27,0.09)	0.31	−0.09 (− 0.26,0.09)	0.34	0.01 (− 0.17,0.19)	0.91	0.03 (− 0.15,0.20)	0.77
Two-parent blended/adoptive	0.01 (−0.14,0.16)	0.87	−0.01 (− 0.16,0.14)	0.93	0.005 (− 0.13,0.14)	0.95	0.02 (− 0.12,0.15)	0.81
Other family types	0.02 (−0.24,0.28)	0.87	0.06 (−0.21,0.32)	0.67	−0.06 (− 0.33,0.22)	0.69	−0.06 (− 0.34,0.22)	0.68
**SSBs (servings/week)**								
**Number of boys/girls**	2113		2100		2003		1996	
Two-parent biological	Reference		Reference		Reference		Reference	
Single-parent	−0.58 (−2.83,1.66)	0.61	−0.53 (−2.77,1.72)	0.65	3.17 (0.89,5.45)	0.006	3.19 (0.91,5.47)	0.006
Two-parent blended/adoptive	3.08 (1.07,5.10)	0.003	3.01 (0.99,5.03)	0.004	−0.33 (−1.99,1.34)	0.70	−0.33 (−1.99,1.34)	0.70
Other family types	2.09 (−1.33,5.50)	0.23	2.01 (−1.48,5.50)	0.26	−0.99 (−4.30,2.33)	0.56	−0.98 (−4.30,2.34)	0.56

Abbreviations: β, Beta coefficient; CI, Confidence Interval; SSBs, sugar-sweetened beverages

Model 1 is adjusted for sex, age, parental education level, number of children and adults in the household

Model 2 is additionally adjusted for BMI z-score

There was also evidence for an interaction with age group (younger vs. older children) for screen time (see Supplementary Table 1): older children from single-parent families and from blended/adoptive families had higher screen times than those from two-parent biological families (β = 3.58 h/week, 95% CI: 1.45–5.72 and β = 1.91 h/week, 95% CI: 0.23–3.59, respectively) but these associations were not significant in younger children (β = 1.16 h/week, 95% CI: − 0.30-2.62 and β = − 0.21 h/week, 95% CI: − 1.40-0.98, respectively, P_interaction_ ≤ 0.001). Results of the analyses stratified by parental education level are depicted in Supplementary Table 2. Interactions with parental education or European regions (Northern vs. Southern countries) were not statistically significant.

### Association between family structures and family rules

Table 4 shows the association between family structure and the odds ratios (OR) for the presence of family rules regarding these behaviours. Both types of non-traditional families, i.e. single-parent and blended/adoptive families were less likely to have rules regarding watching TV and using the computer (OR = 0.62, 95% CI: 0.40–0.94 and OR = 0.66, 95% CI: 0.47–0.91, respectively). There were no associations between family structure and the availability of SSBs during meals or the presence of bedtime routines.

**Table 4:** Association between family structure and the presence of family rules in European families

	Model 1		Model 2	
	OR (95% CI)	*P*-value	OR (95% CI)	*P*-value
**Rules regarding screen time (yes = 1)**				
**Number of families**	4133		4131	
Two-parent biological	Reference		Reference	
Single-parent	0.61 (0.40,0.93)	0.021	0.62 (0.40,0.94)	0.026
Two-parent blended/adoptive	0.66 (0.47,0.91)	0.011	0.66 (0.47,0.91)	0.011
Other family types	0.96 (0.50,1.84)	0.88	0.97 (0.50,1.86)	0.93
**Bedtime routines (yes = 1)**				
**Number of families**	2978		2976	
Two-parent biological	Reference		Reference	
Single-parent	0.88 (0.53,1.45)	0.62	0.89 (0.54,1.46)	0.64
Two-parent blended/adoptive	1.32 (0.83,2.1)	0.24	1.32 (0.83,2.1)	0.24
Other family types	0.75 (0.37,1.5)	0.41	0.74 (0.37,1.5)	0.40
**Availability of SSBs during meals (yes = 1)**				
**Number of families**	4251		4249	
Two-parent biological	Reference		Reference	
Single-parent	0.82 (0.56,1.20)	0.30	0.82 (0.56,1.21)	0.32
Two-parent blended/adoptive	0.80 (0.60,1.07)	0.14	0.80 (0.60,1.07)	0.13
Other family types	1.14 (0.70,1.85)	0.61	1.14 (0.70,1.86)	0.59

Abbreviations: OR, Odds Ratio; CI, Confidence Interval; SSBs, sugar-sweetened beverages

Model 1 is adjusted for adjusted for sex (only girls/only boys/both sexes), age (younger (<12 years) children /older (≥12 years) children / younger (<12 years) and older (≥12 years) children), parental education level, number of children and adults in the household

Model 2 is additionally adjusted for the BMI z-score (mean BMI z-score in case of more than one child in the family)

## Discussion

In this pan-European sample, we showed that children from single-parent families have higher screen times than those from two-parent biological families. In addition, boys but not girls from blended/adoptive families had higher screen times than boys from two-parent biological families. Our results further suggest that the higher screen time observed in such families might be due to less stringent rules regarding screen time in single-parent (and also blended) families as compared to traditional two-parent families. Though family structure was not associated with the frequency of SSB consumption overall, subgroup analyses indicated a sex-specific association: boys from two-parent blended and girls from single-parent families consumed more SSBs in comparison to those from two-parent biological families.

Our finding that screen time is lower in children from two-parent biological families is in agreement with several other studies from non-European populations [14, 25, 26], and the fact that two-parent biological families are more likely to have rules regarding screen time has to our knowledge, not yet been previously reported. Mauskopf et al. [12] reported that children living in two-parent families (married or cohabitating parents) consumed less SSBs than those living with separated or divorced parents. We could not confirm such an association in the full sample, but sex-stratified results revealed interesting differences between boys and girls that both point to the fact that boys and girls from two-parent biological families might have the healthiest behaviour regarding SSB consumption. In contrast to Troxel et al. [27] and Hale et al. [41], we did not observe an association between family structures and sleep duration or bedtime routines, respectively.

Thus, our results together with previous studies suggest that family structure is related to children’s screen time behaviour and SSB consumption. Family structure is likely related to underlying family processes such as family cohesion and belonging to the family, which might influence children’s obesogenic behaviour and wellbeing [42]. The parents’ separation is another family process which might account partly for the worse health outcomes of children and adolescents who had experienced this transition [43, 44]. A transition in the means of entering a relationship has no effect on the health status, though [43]. Apart from general health, family processes might have also an influence on children’s obesogenic behaviours and, in consequence, their weight status. A higher prevalence of obesogenic behaviours, for example an unfavourable diet [45], less physical activity, more sedentary time as well as a higher BMI and a higher risk of obesity [45] was shown in children living in families with low family cohesion. Since parent’s separation leads to increased family conflict, less family closeness [46] and poorer parent child relationship [47], it may be one possible explanation why we observed that children and adolescents from non-traditional families have higher screen times, less rules regarding screen time and a higher intake of SSBs. Further, the wish to avoid conflicts, the huge effort it takes to enforce and supervise screen time rules [48] and missing communication about rules between parents and children [49] might be some of the reasons why we observed less rules regarding screen time in single-parent and blended families compared to two-parent families.

Another explanation may be financial resources, which are unequally distributed among these family structure groups. Two-parent biological families have more financial means than other types of families [50, 51], and higher family wealth in such families is related to eating breakfast regularly [52], less eating while watching television [52], a lower consumption of fast food [52] and a higher participation in organized sports [53]. As the lack of participation in organized sports among children growing up in non-traditional families (single-parent and reconstructed families) is likely attributable to missing financial resources [53], lowering the prices or providing state subsidies may lead to a higher sports participation in non-traditional families [53, 54]. Compared to two-parent families, single-parents work more since they have to compensate for the missing parent [55]. As a consequence, they may have less time to spend with their children or to prepare meals. For instance, American single-parent families have less frequent family meals [26] and, with increasing hours that the single-parent works, they spend less time on eating [56]. In addition, children from single-parent families spend more time in day care and after school care facilities than children from two-parent families [56]. However, an earlier study in the same cohort found little evidence of a relationship between maternal employment and obesity, diet and physical activity [57]. Nevertheless, increased screen time in children from non-traditional families may be due to the increased demands and difficulties experienced by single-parents and to some extend blended families. Furthermore, parents might allow children to watch television so that they would have more time to work and rest [58]. Also, single-parents may not have the financial resources to provide alternative activities [48]. In two-parent blended families, the new step-parent may have different rules for screen time and the previous rules may not be maintained. Golish [59] described several difficulties blended families face regarding parenting style. Firstly, the biological parents feel torn between the loyalty to their child and to the new partner in conflict situations. Secondly, there is a role ambiguity with regard to the step-parent, as he/she might lack the legitimate authority to educate the non-biological child. The non-custodial parent, in contrast, might fear a deterioration of the parent-child relationship by being as strict as the custodial parent and an inconsistency of parenting style may arise. Finally, the step-family might also have a different conflict management style. In addition, children in single-parent and blended families may be regularly moving between households (from one biological parent to the other) which might be another reason why such families might have difficulties in maintaining consistency of household rules. To summarize, rules regarding screen time may be more challenging to execute for single-parent and blended families due to increased time and financial constraints and inconsistencies in parenting styles.

Our findings emphasize the necessity for Public Health actions to support non-traditional families, such as single-parent households. As parents in non-traditional family structures might face more challenges in restricting screen time and the consumption of SSBs through rules, alternative strategies for such family types may be needed. For instance, previous studies have shown that covert restriction - where children are unaware that their consumption pattern is influenced - might be more effective in reducing unhealthy eating habits than overt restriction [60–62]. These covert restrictions, such as not bringing certain foods and drinks to the home, or not placing a TV in the children’s bedroom, may be easier to enforce than rules that the children notice.

Also, since children and adolescents spend a lot of time in schools and day-care centres – especially those from single-parent families -, these institutions have a key role in paving the right path for a healthy development of children and adolescents [63]. One example of such an action is the free school meal service available to all preschool and primary school children in Sweden and Finland, regardless of parental income [64]. Moreover, the improvement of secure cycle paths as well as the establishment of more parks and playgrounds can benefit all children and adolescents [63]. Aside from these structural actions, interventions should involve the whole family to improve the family’s lifestyle behaviour, and family cohesion as well as the children’s and adolescent’s resilience against transitions in the living conditions. In summary, a multilevel approach including each member of the family as well as structural changes is crucial to improve the health behaviour in children and adolescents and their families [65].

The results of this study have to be interpreted with regard to its strengths and limitations. Two-parent biological families were more likely to participate in I.Family than other family types and therefore the number of non-traditional families was lower than in the general population [31]. Furthermore, the family structure could not be assigned to some of the participating families due to missing or incomplete information on kinship and household composition (~ 15%). Therefore, more rare family structure types, such as single father families could not be examined separately. Lastly, we cannot exclude the possibility of (residual) confounding due to imprecise measurement of confounders or unmeasured variables such as (but not limited to) neighbourhood deprivation. This study’s strengths include the large sample size of families with in-depth interviews on kinship and household and administration of several questionnaires regarding obesogenic behaviours and family rules. The analysis of family structures, obesogenic behaviours and family rules in the European context is novel. Children growing up with a single-parent had higher screen times compared to two-parent biological families irrespective of the educational level of the parents; however there was a tendency for this association to be stronger among low/medium educated families. The extent to which education can mitigate behavioural differences between children from single-parent and two-parent households should be addressed by future research.

## Conclusion

In this large and well standardized cohort of European families, we found that children growing up in single-parent families had increased screen times and experienced less stringent rules regarding television viewing and computer usage than children from traditional two-parent biological homes. Children from non-traditional families also had a higher frequency of SSB consumption in a sex-specific manner: girls raised in single-parent families and boys raised in blended/adoptive families had higher SSB consumption frequencies as compared to those raised in two-parent biological families. Importantly, these associations were independent of important confounding factors including parental education level. One underlying reason might be that single parents and parents in blended family structures have more difficulties in implementing rules regarding screen time and SSBs than parents in traditional two-parent family structures. Our findings therefore suggest that additional support and effective strategies for non-traditional families may help to reduce obesogenic behaviours in children growing up in such family types.

## Supplementary information

**Additional file 1 Supplementary Table 1** Association between family structure and obesogenic behaviours in European younger (< 12 years) and older (> = 12 years). **Supplementary Table 2** Association between family structure and obesogenic behaviours in European children, stratified by parental education.

## Data Availability

Due to the high sensitive data collected in children, ethical restrictions prohibit the authors from making the minimal data set publicly available. Data are however available from the authors upon reasonable request and with permission of the Steering Committee on a case-by-case basis. Interested researchers can contact the I.Family consortium (http://www.ifamilystudy.eu/) or the study co-ordinator (ahrens@leibniz-bips.de) to discuss possibilities for data access.

## Abbreviations

BMI: Body Mass Index
CI: Confidence Interval
SSBs: sugar-sweetened beverages
