# Supplementary material for: A cross-sectional study of obesogenic behaviours and family rules according to family structure in European children
Source: Int J Behav Nutr Phys Act. 2020 Mar 5;17:32. doi: 10.1186/s12966-020-00939-2 (PMC7059256; doi:10.1186/s12966-020-00939-2)
Supplement: Supplementary file 1 — Additional file 1 Supplementary Table 1 Association between family structure and obesogenic behaviours in European younger (< 12 years) and older (> = 12 years). Supplementary Table 2 Association between family structure and obesogenic behaviours in European children, stratified by parental education. [file 12966_2020_939_MOESM1_ESM.docx]

**Supplementary Table 1** Association between family structure and obesogenic behaviours in European younger (<12 years) and older (>=12 years)

|  | **Younger children** | | | | **Older children** | | | |
| --- | --- | --- | --- | --- | --- | --- | --- | --- |
|  | Model 1  β (95% CI) | P-value | Model 2  β (95% CI) | P-value | Model 1  β (95% CI) | P-value | Model 2  β (95% CI) | P-value |
| **Screen time (hour/week)** |  |  |  |  |  |  |  |  |
| **Number of children** | 4079 |  | 4059 |  | 2934 |  | 2928 |  |
| Two-parent biological | Reference |  | Reference |  | Reference |  | Reference |  |
| Single-parent | 1.22 (-0.24,2.68) | 0.10 | 1.16 (-0.3,2.62) | 0.12 | 3.76 (1.63,5.9) | 0.001 | 3.58 (1.45,5.72) | 0.001 |
| Two-parent blended/adoptive | -0.19 (-1.38,1.0) | 0.75 | -0.21 (-1.4,0.98) | 0.74 | 1.94 (0.25,3.62) | 0.024 | 1.91 (0.23,3.59) | 0.026 |
| Other family types | -0.37 (-2.5,1.76) | 0.73 | -0.39 (-2.54,1.76) | 0.73 | -0.13 (-3.35,3.09) | 0.94 | 0.12 (-3.13,3.37) | 0.94 |
| **Sleep duration (hours/day)** |  |  |  |  |  |  |  |  |
| **Number of children** | 4179 |  | 4159 |  | 2895 |  | 2889 |  |
| Two-parent biological | Reference |  | Reference |  | Reference |  | Reference |  |
| Single-parent | 0.01 (-0.12,0.15) | 0.85 | 0.02 (-0.11,0.16) | 0.74 | -0.1 (-0.33,0.12) | 0.38 | -0.09 (-0.32,0.13) | 0.43 |
| Two-parent blended/adoptive | 0.05 (-0.06,0.16) | 0.40 | 0.05 (-0.06,0.16) | 0.41 | -0.02 (-0.2,0.15) | 0.79 | -0.03 (-0.2 ,0.15) | 0.77 |
| Other family types | -0.02 (-0.23,0.18) | 0.82 | 0.02 (-0.19,0.22) | 0.87 | 0 (-0.35,0.35) | 0.99 | -0.01 (-0.36,0.34) | 0.95 |
| **SSBs (servings/week)** |  |  |  |  |  |  |  |  |
| **Number of children** | 4075 |  | 4055 |  | 41 |  | 41 |  |
| Two-parent biological | Reference |  | Reference |  | Reference |  | Reference |  |
| Single-parent | 0.93 (-0.75,2.6) | 0.28 | 1.0 (-0.68,2.67) | 0.25 | 2.74 (-22.78, 28.26) | 0.83 | 2.36 (-23.16,27.88) | 0.86 |
| Two-parent blended/adoptive | 1.12 (-0.26,2.49) | 0.11 | 1.08 (-0.3,2.45) | 0.13 | 1.11 (-13.06,15.28) | 0.88 | 0.85 (-13.27,14.98) | 0.91 |
| Other family types | 0.59 (-1.86,3.04) | 0.64 | 0.55 (-1.93,3.04) | 0.66 | No observations |  | No observations |  |

Abbreviations: β, Beta coefficient; CI, Confidence Interval; SSBs, sugar-sweetened beverages

Model 1 is adjusted for sex, age, parental education level, number of children and adults in the household

Model 2 is additionally adjusted for BMI z-score

**Supplementary Table 2** Association between family structure and obesogenic behaviours in European children, stratified by parental education

|  | **Low/middle educated families** | | | | **Highly educated families** | | | |
| --- | --- | --- | --- | --- | --- | --- | --- | --- |
|  | Model 1  β (95% CI) | P-value | Model 2  β (95% CI) | P-value | Model 1  β (95% CI) | P-value | Model 2  β (95% CI) | P-value |
| **Screen time (hour/week)** |  |  |  |  |  |  |  |  |
| **Number of children** | 3524 |  | 3514 |  | 3489 |  | 3473 |  |
| Two-parent biological | Reference |  | Reference |  | Reference |  | Reference |  |
| Single-parent | 3.34 (1.54,5.15) | <0.001 | 3.26 (1.45,5.06) | <0.001 | 2.27 (0.35,4.18) | 0.020 | 2.13 (0.22,4.04) | 0.029 |
| Two-parent blended/adoptive | 1.54 (0.05,3.02) | 0.042 | 1.53 (0.05,3.01) | 0.043 | 0.39 (-1.09,1.86) | 0.61 | 0.35 (-1.12,1.82) | 0.64 |
| Other family types | -0.39 (-2.72,1.94) | 0.75 | -0.28 (-2.63,2.08) | 0.82 | 1.53 (-2.18,5.24) | 0.42 | 1.45 (-2.25,5.15) | 0.44 |
| **Sleep duration (hours/day)** |  |  |  |  |  |  |  |  |
| **Number of children** | 3507 |  | 3498 |  | 3567 |  | 3550 |  |
| Two-parent biological | Reference |  | Reference |  | Reference |  | Reference |  |
| Single-parent | 0.05 (-0.14,0.24) | 0.61 | 0.06 (-0.13,0.24) | 0.55 | -0.19 (-0.37,-0.02) | 0.033 | -0.18 (-0.36,-0.01) | 0.043 |
| Two-parent blended/adoptive | 0.05 (-0.1,0.21) | 0.50 | 0.04 (-0.11,0.2) | 0.57 | -0.04 (-0.18,0.1) | 0.57 | -0-04 (-0.18,0.1) | 0.58 |
| Other family types | -0.11 (-0.36,0.13) | 0.36 | -0.09 (-0.34,0.16) | 0.47 | 0.34 (-0.01,0.7) | 0.055 | 0.35 (-0.002,0.7) | 0.051 |
| **SSBs (servings/week)** |  |  |  |  |  |  |  |  |
| **Number of children** | 2023 |  | 2016 |  | 2093 |  | 2080 |  |
| Two-parent biological | Reference |  | Reference |  | Reference |  | Reference |  |
| Single-parent | 1.04 (-1.3,3.39) | 0.38 | 1.1 (-1.25,3.45) | 0.36 | 0.46 (-1.39,2.31) | 0.63 | 0.52 (-1.34,2.37) | 0.58 |
| Two-parent blended/adoptive | 0.82 (-1.08,2.73) | 0.40 | 0.69 (-1.23,2.6) | 0.48 | 1.91 (0.39,3.42) | 0.014 | 1.93 (0.41,3.44) | 0.013 |
| Other family types | 0.5 (-2.51.3.5) | 0.75 | 0.32 (-2.78,3.43) | 0.84 | -0.63 (-4.16,2.89) | 0.73 | -0.6 (-4.13,2.92) | 0.74 |

Abbreviations: β, Beta coefficient; CI, Confidence Interval; SSBs, sugar-sweetened beverages

Model 1 is adjusted for sex, age, parental education level, number of children and adults in the household

Model 2 is additionally adjusted for BMI z-score
